# Editorial: New challenges in arrhythmology

**DOI:** 10.3389/fmed.2025.1622534

**Published:** 2025-08-14

**Authors:** Martina Nesti, Laura Vitali Serdoz

**Affiliations:** ^1^Electrophysiology, Fondazione Toscana Gabriele Monasterio, Pisa, Italy; ^2^Arrhythmia and Electrophysiology Division, Department of Heart and Lung, Klinikum Fuerth, Fuerth, Germany

**Keywords:** atrial fibrillation, device, mapping, ablation, electrophysiology

This Research Topic presents a cohesive collection of contributions that touch the full translational spectrum of cardiac electrophysiology—from mechanistic exploration and technological advancement to clinical applicability and therapeutic innovation. Together, these studies form a mosaic that not only highlights the dynamism of the field but also offers practical insights for shaping next-generation patient care.

At the heart of these innovations is the aim to personalize therapy improving safety, efficacy, and access to care. This editorial invites readers to consider each article as interconnected pieces that advance our understanding and capabilities in arrhythmia management.

The studies included in this Research Topic represent not only technological progress but a paradigm shift in how we understand and manage cardiac arrhythmias. Device therapy, as demonstrated by Russo et al., is now focused not merely on extending life, but on enhancing quality of life and reducing long-term procedural risk. This indicates a more nuanced, patient-centered approach in clinical decision-making.

Electroanatomical mapping studies by Chen et al. and Drago et al. show how electrophysiology is evolving into the acquisition of a real-time spatial data to predict outcomes and tailor procedures. Their work underscores how technical innovation can deepen physiological insight.

The advent of pulsed field ablation, covered by Zhai et al. and Ma et al., brings safety and specificity into focus. This energy source represents a fundamental change in ablation philosophy—offering cardiac-specific effects with reduced risk to other structures. Such transformation opens new possibilities for treating arrhythmias also in anatomically sensitive locations.

The analysis of arrhythmogenesis, discussed by Hytting et al., Ou et al., and Henson et al., demonstrate how arrhythmias are not purely electrical events but reflections of endocrine, metabolic, and pharmacologic imbalances. These insights advocate for a broader, integrative view in arrhythmia diagnosis and prevention.

Menichetti et al.'s work on cardiac contractility modulation invites readers to reconsider the role of device therapy—not just as an endpoint but as a tool for recovery and transition. This vision opens doors to new therapy for advanced heart failure patients. This editorial is a trip inside articles that collectively point toward the future of cardiac electrophysiology. Readers will find in these studies not only innovation but also a roadmap for what the electrophysiology can become when engineering, clinical insight, and translational science meet.

The reader should not only explore each article, but see the connections among them. This Research Topic reflects a push toward integrated, tailored, and safer care across patient populations. The synergy among mapping, ablation, device therapy, and systemic understanding will improve more and more physicians in a comprehensive taking care of their patients.

